# Editorial: Ecological Consequences of Biodiversity and Biotechnology in Agriculture and Forestry

**DOI:** 10.3389/fpls.2016.00210

**Published:** 2016-02-19

**Authors:** Martin Weih, Andrea Polle

**Affiliations:** ^1^Department of Crop Production Ecology, Swedish University of Agricultural SciencesUppsala, Sweden; ^2^Department for Forest Botany and Tree Physiology, Büsgen-Institute, Georg-August-Universität GöttingenGöttingen, Germany

**Keywords:** agriculture, biodiversity, biotechnology, ecology, forestry, genetically modified organisms, stress resistance, yield

With the increasing recognition of world population feeding and health, global climate change and biodiversity loss, and limited energy resources with fossil fuels calling for alternatives such as biomass crops, the relevance of agriculture, and forestry for human well-being in the future is more than evident. In this context, applications of biotechnological methods including genetic engineering, marker-assisted breeding, clonal propagation of elite trees, etc., are becoming very important, but are frequently debated in the public. Genetically modified organisms were first introduced into commercial agriculture more than two decades ago, and have often led to higher yields but also more flexible and efficient management strategies (Zilberman et al., 2010). Trait manipulation of target organisms and production system components also creates opportunities for improved products obtained with more effective resource utilization and reduced negative environmental impact. Nevertheless, manipulated traits may introduce unforeseen effects on ecological processes. Due to the complexity of agricultural and tree production systems and the different scales involved in the biological studies with genetically modified organisms on one hand and ecological studies targeting ecosystem processes on the other hand, trans- or inter-disciplinary approaches are often needed. The intention of this Research Topic was to highlight the need for integrated approaches in research activities and to bridge research progress within the areas of plant biology, ecology, and ecosystem science. Contributions deal with various aspects of crop/tree biotechnology and diversity for biomass, food and feed production and their ecological consequences.

Important issues in using biotechnology in agriculture and forestry are for example to enhance productivity and stress resistance of crops and trees, mainly due to restricted land area and increasing environmental pressures, and to develop carbon dioxide-neutral production systems for sustainable production of fiber/biomass and biofuel with biotechnological methods (Polle et al., 2013; Polle and Chen, 2015). Along with the production issues, we need to conserve and protect natural diversity and species richness as a foundation of life on earth. With the recognition that increased plant diversity may also increase productivity, especially at low resource input (e.g., Weigelt et al., 2009), novel production systems combining aspects of diversity and biotechnology are emerging.

Biotechnological methods are currently being developed to explore and make better use of the genetic diversity in important crops, as was reported by Nyaboga et al. for cassava cultivars often grown by farmers in east Africa. Pathogens are one of the biggest threats to crop production in many production systems, and modern biotechnology offers excellent possibilities for high-throughput methodologies for the rapid and efficient screening of economically important crop pathogens. The work by Zeng et al. provides a nice example for the development of a biotechnology based methodology for the early detection and quantification of a potentially important plant pathogen, although verification of the methodology in crop plants and under field conditions still remains to be done. Crop products such as grains are often used as feed in animal production, but need to be stored for extended periods for this purpose which implies the increased use of fossil resources for instance for drying the grain. Alternatively, the moist storage of the grain has environmental and also nutritional advantages, and can be facilitated by using appropriate microorganisms with the moist stored grain. An example for this technology is reported by Borling Welin et al. who exploited the microbial diversity of yeast with biotechnological methods to ultimately improve an animal production system in terms of less use of fossil fuels and enhanced nutritional quality of the feed grain.

More direct uses of biotechnology are applied to improve crop and tree management and yield by modification of plant architecture (review by Teichmann and Muhr), to enhance the stress resistance of economically important plants (Aygun and Dumanoglu; Xiao et al.), or to enhance the productivity and stress (drought, pests) resistance of trees in the development of CO_2_-neutral biomass production systems (Hennig et al.; Hjältén and Axelsson). The ecological consequences of biomass production systems need to be evaluated at landscape scale (Bredemeier et al.), and biotechnological methods can be used with advantage to investigate the relationships between genetic diversity in tree plantations and an indicator for biodiversity (here arthropod abundance) as an ecosystem service (Zhang et al.). The latter paper is one of few examples in which serious efforts were made to link genetic diversity of a dominating tree with biodiversity at landscape scale.

The contributions to this Research Topic represent an impressing breath of biotechnology applications in agriculture and forestry. However, keeping in mind that genetically modified organisms have now been used for more than two decades, surprisingly few reports were submitted with a clear focus on the ecological consequences of biotechnology in agriculture and forestry. The poor representation of investigations on ecological consequence assessments is probably indicative of the general paucity of studies linking genetically modified plant traits to ecosystem processes at longer time scales recently pointed out by Kolseth et al. (2015), and illustrates a difficulty when bridging ecological impact assessment and plant breeding: Major targets for ecological impact assessment are quantities at the ecosystem level, while the targets for plant breeding are individual plant traits. Irrespective of the technology of crop/tree improvement used, our knowledge on the mechanistic links between individual plant traits and ecosystem processes is poor and needs to be investigated more in the future (Weih et al.). In this context, Kolseth et al. (2015) noted that biotechnology may provide a unique tool for gaining insights into the links between plant traits and ecosystem processes when integrated into basic ecological research. The contributions to this Research Topic indicate an enormous potential for biotechnology applications to improve agricultural and forestry production systems, but also call for better integration of future research activities bridging the relevant subject areas. A major focus of this research should be on the specific traits of modified organisms and their possible ecological consequences, rather than the technologies used to modify those traits.

## Author contributions

MW drafted the main parts of the text and AP contributed to the writing.

### Conflict of interest statement

The authors declare that the research was conducted in the absence of any commercial or financial relationships that could be construed as a potential conflict of interest.
